# Post-treatment adaptation experiences of women breast cancer survivors: a qualitative study

**DOI:** 10.1007/s00520-026-10683-1

**Published:** 2026-04-17

**Authors:** Nisa Yavuzer Bayrak, Serpil Özcan, Gülcan Bahçecioğlu Turan

**Affiliations:** 1 Department of Nursing, Faculty of Health Sciences, Nevsehir Haci Bektas Veli University, Nevşehir, Turkey; 2 Department of Fundamental of Nursing, Ataturk University, Erzurum, Turkey; 3 Nursing Department, Faculty of Health Sciences, Fırat University, Elazığ, Turkey

**Keywords:** Breast neoplasms, Cancer survivors, Adaptation, Psychological, Coping behavior, Social support, Qualitative research

## Abstract

**Objective:**

This study was conducted to explore breast cancer survivors' experiences with their adjustment process.

**Method:**

The study population included women registered at a university hospital who had completed active treatment (surgery, chemotherapy, and/or radiotherapy) between May and July 2025, including those continuing hormone therapy. The study sample included 20 women who agreed to participate. The criterion sampling method was employed. The research data were collected through 'Introductory Information Form', 'Semi-structured Questionnaire’, and face-to-face individual interviews. Interviews were recorded with a voice recorder, with the participants' permission. The content analysis method was used to evaluate the qualitative data. In the study, the COREQ (Consolidated Criteria for Reporting Qualitative Research) Checklist was used to report qualitative research.

**Results:**

As a result of the analysis of the data obtained from the interviews on the experiences of breast cancer survivors regarding the adaptation process, five themes, namely "Difficulties in the Adaptation Process", "Emotional Experiences", "Coping Strategies", "Change in Lifestyle and Self-Perception" and "Support Mechanisms in the Adaptation Process", and a total of 34 codes for these themes were created.

**Conclusion:**

The findings showed that women experienced physical and emotional challenges during the adaptation process, including fatigue, weakness, limited mobility, and emotional confusion. Coping strategies included appearing strong and relying on trust and gratitude. Women also reported changes in lifestyle, self-perception, and life perspective, with a greater focus on living in the moment and self-care. Family members, especially children and spouses, were identified as the main sources of support.

## Introduction

Breast cancer is the most common type of cancer worldwide and remains a major global health concern. However, advances in diagnosis and treatment have significantly improved survival rates [1]. Despite these improvements, women often face challenges when readjusting to daily life after completing treatment. Recovery requires a multidimensional adaptation process that includes re-engagement in social life and a return to functional roles [2].

Following treatment, women may experience various physical problems that negatively affect quality of life. Common issues include fatigue, pain, limited mobility, hormonal changes, and alterations in body image [2, 3]. In addition to physical difficulties, many women experience emotional challenges such as stress, anxiety, and depression. Fear of cancer recurrence is also frequently reported during the recovery period [4–6].

In addition to physical and emotional recovery after breast cancer treatment, social adaptation is also an essential part of recovery. Changes in social roles and relationships may occur, and women can experience difficulties returning to work or participating fully in social life. Feelings of social isolation may further complicate this adjustment process [7, 8].

Healthcare professionals play a critical role in supporting women’s physical, emotional, and social adaptation after treatment. Nurses, in particular, provide essential guidance by addressing individual needs through care delivered during and after treatment [9, 10]. Previous studies have shown that nursing interventions can improve quality of life in the post-treatment period. Personalized care and telephone follow-up programs have been associated with improvements in emotional functioning and reduced anxiety level [9, 11, 12].

Similarly, nursing interventions focusing on psychological well-being have demonstrated positive effects on depression and anxiety. In addition, supportive practices such as beauty care interventions have been linked to improved self-esteem and quality of life, suggesting that multifaceted nursing approaches contribute to the adaptation process [12–14].

Overall, existing evidence highlights the importance of nurse-led interventions that provide comprehensive and continuous support after treatment. Therefore, gaining a deeper understanding of the physical, psychological, and social challenges experienced by breast cancer survivors is essential for improving the quality and effectiveness of nursing care and for developing interventions that better meet patients’ needs [15]. Although international studies have examined post-treatment adaptation in breast cancer survivors, adaptation experiences may differ depending on cultural and healthcare contexts. In Turkey, sociocultural factors such as strong family support, traditional gender roles, and expectations related to women’s caregiving responsibilities may influence how women experience illness and recovery after treatment. Spirituality and religious coping, which are common in daily life, may also shape coping strategies and the way women make meaning of their experiences. Furthermore, differences in healthcare systems can affect post-treatment experiences. Therefore, understanding adaptation within the Turkish context is important for providing culturally sensitive and needs-based nursing care. In the context of this study, adaptation is understood as a multidimensional and dynamic response to the challenges imposed by illness. It involves not only physical adjustment to bodily changes, but also emotional regulation, cognitive reinterpretation of the illness experience, redefinition of social roles, and spiritual meaning-making. Adaptation does not represent a single outcome; rather, it reflects an ongoing process through which individuals reorganize their lives, reconstruct their sense of self, and integrate illness into their life narratives. Within breast cancer survivorship, adaptation encompasses both vulnerability and resilience as women navigate changes in identity, relationships, and daily functioning. Although some studies have explored breast cancer survivorship in Turkey, qualitative evidence addressing the physical, emotional, social, and spiritual dimensions of adaptation together remains limited. Because post-treatment adaptation is a complex and multidimensional phenomenon shaped by subjective meanings and personal interpretations, quantitative approaches alone may not fully capture women’s experiences. Therefore, a qualitative research design was considered appropriate to explore the depth and contextual nature of adaptation within the Turkish setting. By exploring the lived experiences of women in eastern Turkey, this study aims to provide context-specific insights to support culturally appropriate nursing interventions and survivorship care.

## Methods

### Type of research

This study was conducted as a qualitative descriptive study.

### Study population and sample selection

The population of the study consisted of women who were treated in a university hospital in eastern Turkey between May and July 2025 and completed active treatment (surgery, chemotherapy, and/or radiotherapy) after being diagnosed with breast cancer. Individuals who continued hormone therapy were allowed to participate in the study. Participant recruitment continued until data saturation was achieved. The final sample consisted of 20 women who met the inclusion criteria and agreed to participate. (Inclusion criteria are: Being 18 years of age or older, Having been diagnosed with breast cancer, Having completed surgery, chemotherapy and/or radiotherapy at least one year ago, Continuing hormonotherapy does not prevent participation, Having the ability to speak and understand Turkish, Exclusion criteria are as follows: Those who continue active cancer treatment (chemotherapy, radiotherapy, surgery), Those with cancer recurrence or metastatic stage, Severe cognitive impairment that prevents participation in the interview). The criterion sampling method was used. Women with breast cancer who had completed treatment for at least one year or more were included in the sample. The reason for choosing this criterion was that the women had had enough time to evaluate the effects of the disease and to reflect on the adaptation process retrospectively.

### Data collection tools and features

A Descriptive Information Form consisting of 6 questions (age, marital status, employment status, economic status, time elapsed after completion of cancer treatment, type of treatment received) and 10 semi-structured questions developed by the researchers with the support of Example interview questions included:

“What difficulties did you encounter while returning to daily life after completing treatment?”,

“What emotional experiences did you have during the adaptation period?”, and “How did your relationships with family and friends change after cancer?” These open-ended questions were designed to explore participants’ physical, emotional, social, and existential dimensions of post-treatment adaptation. the literature was used to collect the data.

The semi-structured questions were presented to 7 experts. After receiving expert opinions, four pilot applications were conducted (pilot applications were not included in the analysis), and the semi-structured interview form was finalized.

### Data collection

Data were collected from women who had been treated and survived breast cancer at a university hospital in eastern Turkey between May and July 2025. The data were collected voluntarily after the participants were informed about the purpose and method of the study. The data collection process was conducted by G.B.T. and N.Y.B., nurse academics trained in qualitative research.

Data were collected from the participants through face-to-face individual interviews. Interviews were conducted in a quiet and confidential environment (hospital interview rooms and home environment, with the consent of the participant) where the participants felt comfortable. Before starting the interviews, participants were informed about the purpose of the study and the data collection method. Then, after obtaining the written/verbal consent of the participants, the introductory information form and semi-structured questions were administered to the participants. Each interview lasted approximately 10–20 min. Although relatively brief, participants had previously experienced and reflected on their illness and post-treatment adjustment process, which enabled them to express their experiences clearly and meaningfully. When necessary, follow-up and clarification questions were asked to deepen and elaborate on responses. Interviews were audio-recorded with participants’ permission. Only the researcher and the participant were present during the interviews. Each participant was assigned a code (P1, P2, …P20) to ensure confidentiality.

Data collection and preliminary analysis proceeded concurrently. As interviews progressed, recurring patterns and thematic repetition became evident, and no substantially new conceptual insights emerged. Based on this observed stability in coding and theme development, data collection was concluded with 20 participants.

### Data evaluation

After the research design was determined, the data were analyzed in line with the COREQ (Consolidated Criteria for Reporting Qualitative Research) Checklist guide developed for reporting qualitative research [16].

After the interviews were completed, the data recorded on the voice recorder were transferred to the computer with the code names of the participants. It was then transcribed into written text by the researchers. The data obtained in the study were analyzed by the content analysis (inductive) method. The data were analyzed separately by hand coding by the researchers (G.B.T., S.Ö., and N.Y.B.), who were trained in qualitative research. Then, the analyses made by the researchers were compared and finalized through a consensus. The content analysis method was conducted in four stages [17].

- Phase 1: Coding the data: In this stage, transcripts were read repeatedly, and meaningful units were identified and labeled with codes derived directly from participants’ statements.
- Phase 2: Creation of themes: Codes with conceptual similarities were compared and grouped together to form subcategories and broader themes representing common patterns in the data.
- Phase 3: Organizing data according to codes and themes: The coded data were reorganized under relevant themes to ensure coherence and consistency across the dataset.
- Phase 4: Interpretation of findings: Finally, themes were interpreted in relation to the research aim, and meanings were derived by examining relationships among themes and participants’ experiences.

### Research ethics

Ethical approval was obtained from the Ethics Committee of Fırat University Non-Interventional Research (Date: 10.10.2024; Approval No: 2024/13–32), and institutional permission was obtained from the institution where the research was conducted. Participants were informed that the data would only be used for research purposes and that they could withdraw from the study at any time. Before starting the face-to-face interviews, written and verbal informed consent was obtained from the participants for their voluntary participation. The entire research process was conducted following the principles of the Declaration of Helsinki.

## Results

The study involved 20 women who had survived breast cancer. The women's ages ranged between 34 and 55. Nineteen of the women were married, 14 were unemployed, 14 had moderate economic status, and 18 had 1–5 years after the completion of breast cancer treatment. 7 of the women received all three treatments of Chemotherapy, Radiotherapy, and Surgery (Table 1).

**Table 1:** Demographic characteristics of women breast cancer survivors

Participant	Age	Marital Status	Employment status	Economic situation	Time elapsed after completion of cancer treatment	Type of treatment received
1	40	Married	Yes	Average	1–5 years	Chemotherapy, Surgical Treatment
2	49	Married	Yes	Average	1–5 years	Chemotherapy, Hormone Therapy, Surgical Treatment
3	47	Married	No	Average	1–5 years	﻿Chemotherapy, Radiotherapy, Hormone Therapy
4	44	Married	Yes	Good	6–10 years	﻿Chemotherapy, Radiotherapy, Hormone Therapy, Surgical Treatment
5	46	Married	Yes	Average	1–5 years	Chemotherapy, Radiotherapy, Surgical Treatment
6	48	Married	No	Average	1–5 years	Chemotherapy, Radiotherapy, Hormone Therapy, Surgical Treatment
7	41	Married	No	Average	1–5 years	Chemotherapy, Radiotherapy, Surgical Treatment
8	37	Married	No	Average	1–5 years	Chemotherapy, Radiotherapy, Surgical Treatment
9	37	Married	No	Average	1–5 years	Radiotherapy, Hormone Therapy, Surgical Treatment
10	44	Married	No	Good	1–5 years	Chemotherapy, Radyotherapy
11	45	Married	Yes	Good	1–5 years	Radiotherapy, Hormone Therapy
12	42	Married	No	Good	1–5 years	Chemotherapy, Radiotherapy, Surgical Treatment
13	44	Married	No	Average	1–5 years	Chemotherapy, Radiotherapy, Hormone Therapy, Surgical Treatment
14	46	Married	No	Average	1–5 years	Chemotherapy, Radiotherapy, Surgical Treatment
15	55	Married	No	Average	1–5 years	Chemotherapy, Surgical Treatment
16	35	Married	No	Average	1–5 years	Chemotherapy, Surgical Treatment
17	50	Married	No	Average	6–10 years	Chemotherapy, Radiotherapy, Surgical Treatment
18	34	Married	Yes	Good	1–5 years	Chemotherapy, Radiotherapy, Surgical Treatment
19	42	Married	No	Average	1–5 years	Chemotherapy, Surgical Treatment
20	44	Single	No	Poor	1–5 years	Chemotherapy, Surgical Treatment

Findings indicated that post-treatment adjustment was experienced as a dynamic and interconnected process rather than isolated events. Women initially encountered physical difficulties, which were accompanied by emotional responses. In response to these challenges, they developed various coping strategies. Over time, these experiences contributed to changes in lifestyle and self-perception, while support mechanisms played a facilitating role throughout the adjustment trajectory.

As a result of the analysis of the data obtained from the interviews conducted on the experiences of breast cancer survivors regarding the adaptation process, 5 themes and 34 codes were formed as "Difficulties in the Adaptation Process", "Emotional Experiences", "Coping Strategies", "Change in Lifestyle and Self-Perception" and "Support Mechanisms in the Adaptation Process" (Table 2).

**Table 2:** Themes and codes obtained from the interviews

Themes	Codes	Participant	n
Difficulties in the Adaptation Process	Fatigue/weakness	1,3,4,6,7,9,11,14,15,16,17,18,19,20	14
	Limited physical movement	2,8,9,11,12,14,15,16,17,18,20	11
	Numbness/pain/swelling in the arm	1,4,3,5,6,8,9,15,18,20	10
	Difficulty with daily tasks	2,3,7,8,9,13,14,16,17,18	10
	Social unconsciousness	1,3,4,5,6,8,9,10,13,14	10
	Muscle/bone pain	1,3,9,10,11,13,17,19,20	9
	Inability to lift heavy objects	3,7,8,9,18	5
Emotional Experiences	Emotional turmoil	1,2,3,6,7,9,11,16,18	9
	Burnout	1,6,7,8,9,14,15,20	7
	Feeling powerless/hopeless	1,5,6,14,15,17,20	7
	Feeling sad about losing your body image	2,7,14,15,16,17,18	7
	Strengthening family/friendship bonds	1,2,3,8,9,11,13,15,19,20	7
	Feeling powerful	8,12,13,16,19	5
	Irritability/Stress	3,4,6,7,10	5
Coping Strategies	Trying to appear strong	1,2,4,8,11,14,16,17,18,19	10
	Tawakkul/Thankfulness	2,3,6,7,8,9,11,14,15,17	10
	Religious rituals	2,7,8,9,15,16,17,18	8
	Not thinking about the disease	1,3,6,7,9,11,13,14	8
	Social distancing	6,11,12,13,16,18,20	7
	Self-motivation	3,6,11,12,16,18	6
	Avoiding introspection	1,3,6,7,13,18	6
	Spending time with family/friends	7,8,9,10,14,18	6
	Being busy at work	1,2,5,11,18	5
Change in Lifestyle and Self-Perception	The desire to live in the moment	1,2,7,8,9,11,16,17,19,20	10
	Decreased quality of life	5,7,8,15,16,17,18,19,20	9
	Being positive/hopeful	4,5,6,10,11,13,16,18,19	9
	Self-discovery/valuing	7,8,12,13,15,16,17,18,19	9
	Distancing from society/family	6,7,10,12,15,18	6
Support Mechanisms in the Adaptation Process	Children	1,5,7,8,11,14,15,17,18,19,20	11
	Husband	2,4,7,9,11,12,14,16,18,19	10
	Other family members/friends	2,4,5,10,11,12,13,16,17,19	10
	Public awareness	16,17,18,19,20	5
	Mother	2,7,13,16,18	5
	Self-support	3,6,12,14,17	5

### Theme 1. Difficulties in the adaptation process

Seven codes were identified for the theme of difficulties experienced in the adaptation process (Table 2). These physical challenges were not experienced merely as isolated symptoms but as persistent limitations that reshaped women’s sense of bodily capacity and independence in daily life. For many participants, physical difficulties represented the initial and most visible dimension of post-treatment adjustment. After surviving breast cancer, the most common problems that women experience during the adaptation process are fatigue, weakness, and limitation in physical movement. Women stated that the most important reasons for the limitation in physical movement were the inability to use the arm on the side of the surgical operation and the numbness, swelling, and pain while using it. In addition, the women stated that they had difficulties in doing their daily work and housework, and that they had problems due to the insensitivity or unsympathetic attitudes of society, due to insufficient awareness of the disease. They also emphasised that they suffered from muscle and bone pains and that they had problems such as not being able to lift heavy objects, especially with the arm on the side that had undergone surgery. Some participants' views on the codes given in Theme 1 are as follows:

- P1: “I get tired very easily. I have a lot of muscle and bone pain, as well as tingling and numbness in my arm. This disease has become commonplace for healthcare workers, and they don't act sensitively. Society isn't very conscious anyway; they don't see the breast as an organ.”
- P9: "For example, it affects me when I lift water or something heavy."
- P14: "I had difficulty because my breast was removed… I couldn't go to the supermarket, I was weak, and I had a lot of difficulty walking."

### Theme 2. Emotional experiences

Seven codes were identified for the theme of emotional experiences (Table 2). Emotional responses reflected an internal struggle between vulnerability and resilience. Participants described both distressing feelings such as loss and helplessness, and emerging perceptions of strength, suggesting that emotional adjustment was characterized by coexistence of fragility and growth. Women stated that they experienced the most emotional turmoil in the process after surviving breast cancer, that they sometimes had to suppress their emotions, and that they were hesitant to show their sadness or joy. In addition, women reported feeling exhausted, empty, and powerless, and that they had lost hope for the future. They emphasised that the loss of their breasts and hair had caused them sadness, that family and friend ties had strengthened in the period following recovery from the illness, that some women felt stronger, and that they had also experienced irritability and stress. Some participants' views on the codes given in Theme 2 are as follows:

- P2: "I am experiencing such an uneasiness spiritually. I am experiencing sadness, loss, I wish I had my chest… In my relationship with my husband, I discovered aspects of him that I had not realised before; I saw that he was unexpectedly compassionate, naive, and made me feel that he cared for me."
- P8: "I felt exhausted. Then a strength came from God, and I was grateful. As my spiritual strength increased, I felt stronger."
- P10: "I'm too emotionally irritable."
- P11: "Emotionally, sometimes I feel happy, sometimes unhappy. I experience a lot of emotional complexity. I feel as if some of my feelings have disappeared."

### Theme 3. Coping strategies

Nine codes were identified for the theme of coping strategies (Table 2). Coping strategies functioned as adaptive mechanisms through which women attempted to regain control over their lives. These strategies ranged from cognitive avoidance to spiritual reliance and active engagement in daily roles, reflecting both protective and transformative dimensions of adjustment. Women stated that they used various strategies to cope with the difficulties and changes in their emotional state after surviving cancer. It was determined that the most common coping methods they used were efforts to appear strong, as well as trust and gratitude. They also reported engaging in religious rituals such as praying, reading the Qur'an, trying not to think about the illness, and socially distancing themselves from people who reminded them of their illness, showed no understanding, or triggered their sadness. The women also tried to maintain their emotional well-being by motivating themselves, avoiding introspection to avoid recalling negative emotions and experiences, having fun with family members and friends, and returning to and adapting to working life. Some participants' views on the codes given in Theme 3 are as follows:

- P3: "I don't worry about this disease anymore. I leave it to God. What God says is what God says."
- P4: "You don't have the luxury of feeling weak because I have children, and my children were still in primary school. So I had to be strong."
- P5: "I gave myself to my work; work came to me."
- P17: "I cared less about my husband, I didn't care much. I used to feel sad, cry, and serve him more. Now I don't do anything. I take strength from Allah; praying, praying, and reading the Qur'an relaxes me and makes me feel stronger and happier."
- P18: "I prayed a lot to feel strong again. I spent time with the people I love. I started playing games with my daughter. I spent a lot of time with her. I took some people out of my life, and I didn't include too many people in my life. I stopped seeing people who I believed were hurting me, who made me uncomfortable, who didn't do me good, who didn't motivate me."

### Theme 4. Change in lifestyle and self-perception

Five codes were identified for the theme of change in lifestyle and self-perception (Table 2). Changes in lifestyle and self-perception indicated a redefinition of identity following illness. Participants’ narratives suggested a shift from externally focused caregiving roles toward increased self-awareness, self-valuation, and present-oriented living. It was determined that there were various changes in the lifestyle, life view, and self-perception of women after surviving breast cancer. Women emphasised that they did not plan for the future after surviving cancer and preferred to live in the moment instead of hoping for the future. They also stated that they found themselves, discovered themselves, cared more about themselves, and became more positive and hopeful toward life. Some of the women also said that their quality of life had decreased and that they had become distant from society and their family members. Some participants' views on the codes given in Theme 4 are as follows:

- P6: "I was always a person who looked at life with hope. I did not give up hope in God again. It was as if I distanced myself from people. I did not feel good in crowded environments."
- P7: "My quality of life has really decreased a lot. I preferred to get away from everyone. After this disease, I learnt to take care of myself and not to care about anyone else. I learnt to live for myself. I want to live only for myself now."
- P16: "I don't want to make too many plans and tire my mind. I want to continue my life with what I have now. After breast cancer, I pay more attention to myself. In the past, I used to run for my husband and children and keep myself in the background. But the disease reminded me of myself."
- P19: "I would like to live calmly and happily, not chasing big dreams. I see myself as stronger now, as someone who knows herself and cares about herself."

### Theme 5. Support mechanisms in the adaptation process

Six codes were identified for the theme of support mechanisms in the adaptation process (Table 2). Support mechanisms operated as stabilizing resources throughout the adjustment trajectory. Social relationships did not merely provide assistance but actively influenced how women interpreted and managed their post-treatment experiences. Women stated that they received the most support from their children, spouses, other family members, or friends in the process after surviving cancer. In addition, they said that they got through the process more easily with the advice and understanding of their relatives who are familiar with the disease. Some of the women emphasised that they received support from their mothers and themselves. Some participant views on the codes given in Theme 5 are as follows: Some participant views on the codes given in Theme 5 are as follows:

- P12: "The people who helped me the most in my breast cancer recovery process were my family, my husband, and my friends. I focused completely on myself to feel strong."
- P13: "I mean, if I got better today, I got better thanks to my mum. My mum looked after me. I mean, she did not sleep until the morning."
- P15: "My daughters looked after me well when I was ill. My husband did not help me much."
- P20: "Everyone is aware of everything and knowledgeable; they constantly tell me what to do and what to eat and try to guide me…. My children have supported me the most in this process."

## Discussion

This study examined how women who survived breast cancer understood and experienced their return to daily life after completing treatment. The results showed that this process is not a simple or linear return to “normal.” Instead, adaptation continues over time and changes according to physical changes in the body, emotional responses, social responsibilities, and cultural expectations. The five themes identified in the study suggest that adjustment after breast cancer should be seen as an ongoing process in which women continuously redefine their identity and find new meaning in their experiences, rather than as a fixed or completed stage of recovery.

Physical challenges emerged as one of the most prominent aspects of participants’ experiences. Fatigue, pain, limitations in mobility, and functional difficulties were described as persistent conditions that shaped everyday routines and restricted independence. These findings are consistent with previous studies reporting long-term fatigue and physical dysfunction among breast cancer survivors, even years after diagnosis and treatment completion [18–31]. The persistence of such symptoms suggests that physical recovery does not necessarily coincide with the end of medical treatment. Within the cultural context of eastern Turkey, where women are often primary caregivers, reduced physical capacity threatened valued identities related to femininity and productivity. Moreover, insensitive attitudes from society and healthcare settings intensified adaptation challenges, showing that recovery is shaped not only by physical changes but also by social recognition.

Emotional experiences represented another central dimension of post-treatment life. Participants described emotional fluctuations including sadness, fear, irritability, hope, and strengthened interpersonal bonds. These findings align with existing literature demonstrating that emotional adjustment after breast cancer often involves coexistence of relief and anxiety, particularly related to fear of recurrence and long-term treatment effects [32–40]. These emotional responses were not contradictory; rather, they coexisted and reflected a continuous negotiation between feeling vulnerable and feeling strong. Some participants reported suppressing their emotions or avoiding openly expressing sadness and even joy, indicating that managing emotions became part of the adaptation process. In cultural settings where women are expected to remain emotionally stable for their families, appearing strong may serve as a protective strategy, but it can also create additional emotional strain. Changes in body image due to breast and hair loss were described not only as physical alterations but as deeply distressing experiences that affected self-esteem, intimacy, and perceptions of womanhood. At the same time, for some women, the illness led to greater emotional closeness and a redefinition of relationships, showing that emotional adjustment involved both loss and meaningful relational change.

Participants also described a variety of coping strategies used to manage the challenges of post-treatment life. Spirituality, gratitude, family engagement, maintaining activity, and cognitive avoidance strategies were frequently mentioned. Consistent with previous research, spiritual and relational coping appeared to provide emotional comfort and a sense of meaning [41–44]. At the same time, strategies such as avoiding thoughts about illness or distancing from social environments indicate the presence of more internally oriented coping responses that are less frequently emphasized in the literature. Spirituality became an important way for many women to interpret their suffering and uncertainty, helping them find meaning in the illness experience. At the same time, responsibilities such as motherhood shaped coping behaviors, as some women felt they had to stay strong for their children. Strategies like setting boundaries and distancing themselves from certain people also suggest that adaptation involved reshaping social relationships to protect emotional well-being, rather than simply enduring the effects of illness.

Changes in lifestyle and self-perception constituted another important thematic area. Participants described living more in the present, reevaluating priorities, and developing greater self-awareness, while also reporting decreased quality of life and occasional social withdrawal. Similar changes have been described in earlier studies as part of the process of identity reconstruction that occurs during survivorship. In other words, after completing treatment, individuals often begin to reassess who they are, how they see themselves, and how they relate to others [45–51]. Rather than reflecting a uniform positive transformation, the findings demonstrate that lifestyle changes may involve both growth-oriented and challenging experiences occurring simultaneously. This duality emphasizes the importance of understanding adjustment as a complex and individualized experience rather than a single-directional progression.

Support mechanisms, particularly relationships with children, spouses, and family members, were identified as central sources of emotional and psychological strength. Consistent with earlier research, family support appeared to facilitate coping and emotional recovery [52–55]. Participants particularly highlighted their children as a powerful source of motivation and emotional strength during the post-treatment period. For many women, thinking about their children’s future and their responsibilities as mothers gave them a reason to endure physical and emotional difficulties. This emphasis on children shows that the experience of survivorship does not occur in isolation. Instead, it is deeply embedded in family relationships and shaped by ongoing roles and responsibilities within the household. Therefore, survivorship should be understood not only as an individual recovery process, but also as a relational experience that unfolds within the broader context of family life and dynamics.

Overall, the findings contribute to the literature by offering a descriptive understanding of how multiple dimensions of life after treatment coexist and interact. Within the analytic framework used in this study, the themes should not be interpreted as sequential phases of an adjustment trajectory but rather as interconnected experiential domains. This perspective supports conceptual alignment between the analytic approach, terminology, and interpretation of results and emphasizes the diversity of survivor experiences following breast cancer treatment.

## Limitations of the study

This research was conducted over a period of time with only women who had completed breast cancer treatment at a university hospital in eastern Turkey and were breast cancer survivors. Therefore, the findings only represent the experiences, feelings, and perceptions of the women in this sample. Since the study was conducted with a qualitative method and criterion sampling, the results cannot be generalized to different socio-cultural structures, geographical regions, or women receiving treatment in health institutions.

## Conclusion and recommendations

This study examines the experiences of breast cancer survivors in adapting to the disease process from a multidimensional perspective, revealing the challenges they face and the coping strategies they employ at physical, emotional, psychosocial, and spiritual levels. The findings indicate that physical issues such as rapid fatigue, physical limitations, pain, and difficulties in performing daily tasks are commonly experienced during the adaptation process. Additionally, emotional challenges such as emotional turmoil, burnout, body image loss, and hopelessness are also prominently highlighted.

The most common strategies used by participants to cope with these challenges were listed as trying to appear strong, resignation/gratitude, religious rituals, and trying not to think about the illness. In addition, social support and stronger family ties played an essential role in helping some participants better manage the process.

The study also revealed significant changes in lifestyle and self-perception after illness. Trends such as a desire to live in the moment, a decline in quality of life, efforts to be positive, and self-esteem were notable among participants. Finally, while children, spouses, and close family members emerged as the strongest support mechanisms during the adaptation process, turning to one's internal resources was also identified as an important coping mechanism.

The findings reveal that individuals need to be supported in their adjustment process with a holistic approach, emphasising the importance of interventions that go beyond physical symptoms and address emotional, social, and spiritual needs.

Based on the findings of this study, the following recommendations can be made: Considering that participants experience both physical and emotional difficulties during the adaptation process, individualized counselling and rehabilitation programs should be designed. Given the emotional challenges faced, psychological support services should be accessible at all stages of the process. In addition, spiritual counselling should be supported for individuals who value religious/spiritual coping methods. Family members and close friends are an essential source of support during the adaptation process. Therefore, informative training and guidance services should be provided to family members. Considering that participants often complain about social ignorance, it is essential to raise awareness and empathy in society through public awareness campaigns.

## Data Availability

The data supporting this study’s findings are available from the corresponding author upon reasonable request.

## Declarations

### Clinical trial number

Bot applicable.
